# Surgical Outcome of Chronic Pulmonary Aspergilloma: An Experience from Two Tertiary Referral Hospitals in Addis Ababa, Ethiopia

**DOI:** 10.4314/ejhs.v30i4.7

**Published:** 2020-07-01

**Authors:** Berhanu N. Alemu

**Affiliations:** 1 Cardiothoracic Unit, Department of Surgery, School of Medicine, College of Health Sciences, Addis Ababa University, Ethiopia

**Keywords:** Pulmonary Aspergilloma, Cavernostomy, Hemoptysis

## Abstract

**Background:**

Surgical management of pulmonary aspergillosis is challenging and controversial. This study is designed to assess the clinical profile, indications and surgical outcome of Pulmonary aspergilloma.

**Methods:**

A retrospective cross-sectional analysis of 72 patients who underwent pulmonary resection for pulmonary aspergilloma over the period from November, 2014, to November, 2019 was done. Data on demographic, clinical and surgical out come were retrieved. Analysis was done using SPSS version 23. Chi-square test was used to assess for significance of the association between variables and surgical outcome.

**Results:**

There were 46(63.9%) males and 26(36.1%) female patients with a mean age of 35.2+/-11.6 years (Range 16– 65 years). All patients were previously treated for tuberculosis. Cough, hemoptysis, and shortness of breath were the main symptoms identified. A ball of fungus together with the surrounding lung was removed. Accordingly, 32(44,4%) lobectomies, 12(16.7%) pneumonectomy, 7(9.7%) Bi-lobectomy, and 21(29.2%) cavernostomy were done. Intraoperative and Postoperative complications were seen in 8(11.1%) and 21(29.1%) patients respectively. Major morbidity encounters included massive intraoperative blood loss, prolonged air leak, empyema, air space, bronchopleural fistula, and wound infection. The hospital mortality was 3(4.2%) and the average hospital stay was 14.8days. Postoperative complications were evaluated for the difference in socio-demographic characteristics and other variables and a statistically significant difference was detected only for the location of aspergilloma, site of the lung involved and type of surgery done. (P-value =0.05.)

**Conclusion:**

Pulmonary resection done for pulmonary aspergilloma showed favorable outcomes when done with good patient selection, meticulous surgical techniques, and good postoperative management. However, its long term outcome and role of antifungal treatment as adjunctive therapy for surgical resection need further investigation.

## Introduction

Pulmonary aspergilloma (PA) is a ball of fungus that is composed of Aspergillus hyphae, fibrin, mucus, and cellular debris within a pulmonary cavity (1). It usually arises within a preexisting pulmonary cavity that has become colonized with Aspergillus spp (2). The most common underlying pathology that predisposes to PA includes pulmonary tuberculosis, chronic obstructive pulmonary disease (COPD), prior pneumothorax with associated bulla and fibrocavitary sarcoidosis (3). Pulmonary cavities of ≥ 2cm which are left after the treatment of pulmonary tuberculosis have about 20% chance of developing PA (4).

Studies were done using modeling to estimate the global burden of PA as a sequel of pulmonary tuberculosis and showed in the range of =1 case/100,000 populations in western Europe and the USA to 42.9/100,000 population in Democratic Republic of Congo and Nigeria (5). Reflecting on the high frequency of pulmonary tuberculosis, Ethiopia has an estimated rate of 14.5 cases/100,000 population (6).

The natural history of PA has been poorly studied. So far, there appears to be no consistent variable that helps to predict its outcome (7). The course of the disease is extremely variable, ranging from undergoing spontaneous lysis (710%) to causing severe hemoptysis (8). In up to 30% of patients, minor hemoptysis can go on to develop life-threatening hemoptysis (9). Besides, the severity of hemoptysis is also not related to the size, the number of aspergillomas nor to the underlying lung disease (10).

At present, there is a considerable controversy about the optimal treatment for PA, primarily because the natural history of the lesion is not well defined. Moreover, high morbidity and mortality have been reported from some surgical series (11–13). However, the current globally accepted mainstay of treatment is surgery and medical options have shown limited role. In light of the newer available anti-fungal agents, treating with drugs, though disappointing, requires further investigation.

The purpose of this study was to evaluate the results for all patients who underwent pulmonary resection at Tikur Anbessa Specialized Hospital (TASH) and Menillik 2^nd^ Hospital done for 72 consecutive patients with PA.

## Patients And Methods

A retrospective review of medical records and theatre operation register notes of patients operated for PA at Menillik 2^nd^ and Tikur Anbessa Specialized Hospital (TASH) was done. Both hospitals are tertiary referral and teaching hospitals found in Addis Ababa, Ethiopia.

All patients diagnosed to have PA that underwent surgical treatment throughout 5 years (between Nov 30, 2014 – Nov30, 2019) were included. Socio-demographic variables, underlying previous diseases, symptoms, affected lobe of the lung, radiographic findings, indications, and types of surgery, intra-operative and postoperative complications, mortality, and the outcome of surgical interventions were analyzed. Most patients were referred for surgery from other regional hospitals after long-term and repeated treatment of pulmonary tuberculosis. Preoperative assessment and diagnosis of PA were done based on clinical history, physical examination, blood urea nitrogen (BUN) and creatinine, Chest Radiography, and computed tomography of the chest. Pre-operatively for selected patients, pulmonary function tests and echocardiography assessment of left and right ventricular function and possible pulmonary hypertension (if present is a contraindication for surgery) were performed. Immune-diffusion tests or sputum culture for fungus is not routinely done.

Surgical resection was considered for patients with symptoms that justify operations like recurrent hemoptysis, recurrent pneumonia, shortness of breath (SOB) with localized ‘aircrescent’ lesions on CT-Scan of the chest with local destruction of the lung and a good pulmonary reserve. Patients with poor pulmonary reserve or high risk to undergo thoracotomy, and those with concomitant active pulmonary tuberculosis were recommended to have conservative medical treatment.

All patients were operated under general anesthesia. Based on the availability, either a single or double-lumen endobronchial intubation was done. Posterolateral thoracotomy was used to access the thoracic cavity. The choice of resection (cavernostomy, lobectomy, bilobectomy or pneumonectomy) is usually based on the general condition of the patient and the extent of the affected lung. After surgery, most of the patients were extubated in the operating room and sent to the Intensive Care Unit (ICU). Some patients required ventilator support. Routine ICU or postoperative treatment protocol was followed during their hospital stay. Follow-up was subsequently done in the Cardiothoracic surgical referral clinic with serial chest X-rays.

A structured questionnaire was used to collect relevant data from patients’ medical records and operation theater registry logbooks. Data was collected by final year surgery residents. Treatment success for this study was defined as a patient operated for PA and fully improved with no immediate complications seen during their hospital stay or subsequent follow-up visit to the cardiothoracic surgical referral clinic. Data was then cross-checked for completeness, accuracy, and consistency before entry to a software program. Descriptive statistics as percentages, mean, and ranges were computed. Data were expressed as mean ± standard deviation (SD) for the variance of a normal distribution or as the median for those with non-normal distribution. Characteristics of the study subgroups were compared using Mann-Whitney *U*-test, for continuous variables, and Pearson Chi-Square test for categorical variables. The differences in postoperative complications were assessed by univariate analysis. Variables with significance on univariate analysis and previously known risk factors like age, sex, the severity of hemoptysis, past-history of tuberculosis, affected side of the lung and type of surgery done (14–16) were evaluated by multivariate analysis with stepwise backward elimination method to determine the independent predictors of postoperative complications. Statistical analyses were performed using SPSS 23.0 (IBM Corporation, Armonk, NY, USA). For all tests, a value of P=0.05 was considered significant.

## Results

### Socio-demographic profile

Out of the 72 patients included in the study, there were 46(63.9%) males and 26(36.1%) females with male-to-female ratio of 1.8:1. The mean age affected was 35.2 +/−11.6 years (range 16–65). The age group 25–49 years, 53(73.6%) was predominantly affected. Besides, the majority of patients came from urban areas, 43(59.7%), and were married, 43(59.7%) (Table 1).

**Table 1:** Analysis of demographics, Clinical features and its relation to Postoperative complications done at Tikur Anbessa Specialized Hospital and Menilik the 2nd Hospital from Nov 30, 2014 – Nov30, 2019 1Pearson Chi-Square, *reference group used to calculate AOR

	Postoperative Complications					
	No(%)	Yes(%)	Total (%)	P-Value^1^	AOR	95%CI
**Demographics**						
Sex – M	32(64)	14(63.6)	46(63.9)	0.976	1.016	0.358–2.883
- F	18(36)	8(36.4)	26(36.1)			
Age (years)	35.2 +/−11.6			0.445	1.016	0.975–1.059
Age (Years)						
- = 25*	8(16)	3(13.6)	11(15.3)	0.647	1.259	0.470–3.373
- 25–49	37(74)	16(72.7)	53(73.6)			
- >49	5(10)	3(13.6)	8(11.1)			
Address
- Urban	33(66)	10(45.5)	43(59.7)	0.105	2.329	0.837–6.480
- Rural	17(34)	12(54.5)	29(40.3)			
Marital status						
- Single*	16(32)	11(50)	27(37.5)	0.478	1.385	0564–3.404
- Married	32(64)	11(50)	43(59.7)			
- Widowed	2(4)	0(0)	2(2.8)			
**Clinical features**						
Cough	50(100)	22(100)	72(100)	–	–	–
Pain	35(70)	15(68.2)	50(69.4)	0.877	0.918	0.311–2.710
Hemoptysis						
- Mild*	29(58)	11(50)	40(55.6)	0.917	1.033	0.560–1.905
- Moderate	9(18)	7(31.8)	16(22.2)			
- Severe	12(24)	4(18.2)	16(22.2)			
Fever	17(34)	9(40.9)	26(36.1)	0.575	1.344	0.479–3.771
SOB	23(46)	13(59.1)	36(50)	0.308	1.696	0.614–4.682
Tuberculosis	50(100)	22(100)	72(100)	–	–	–
Diabetes Mellitus	1(2)	1(4.5)	2(2.8)	0.556	2.333	0.139–39.08
HIV - Positive*	1(2)	1(4.5)	2(2.8)	0.432	0.685	0.267–1.760
- Negative	11(22)	6(27.3)	17(23.6)			
- Unknown	38(76)	15(68.2)	53(73.6)			
Deranged V/S	8(16)	1(4.5)	9(12.5)	0.205	0.250	0.029–2.133
Pallor	12(24)	5(22.7)	17(23.6)	0.907	0.931	0.283–3.061

### Diagnosis

The diagnosis of PA was based on clinical features and diagnostic investigations. Cough, 72(100%), and hemoptysis, 72(100%), were the primary symptoms reported in all patients. Other symptoms were pain, 50(69.4%), fever, 26(36.1%), and SOB, 36(50%). The majority of the patients had mild hemoptysis, 40(55.6%). Only 16(22.2%) had a history of severe hemoptysis.

All patients were treated for pulmonary tuberculosis, and 49(68%) of them were treated for more than one time. The time between the first incidence of pulmonary tuberculosis and subsequent development of aspergilloma was variable (range 1–20 years). Concomitant illnesses identified were diabetes mellitus, 2(2.8%), and HIV infection, 2(2.8%). The vital sign was deranged in 9(12.5%) patients, and 17(23.6%) patients had Pallor (Table 1).

**Figure 1:**
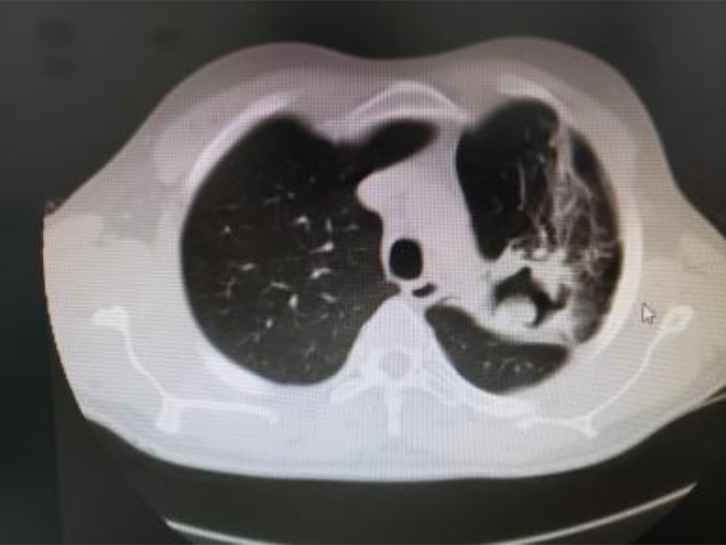
Left upper lobe cavity with internal soft tissue density with surrounding fibrosis

**Figure 2:**
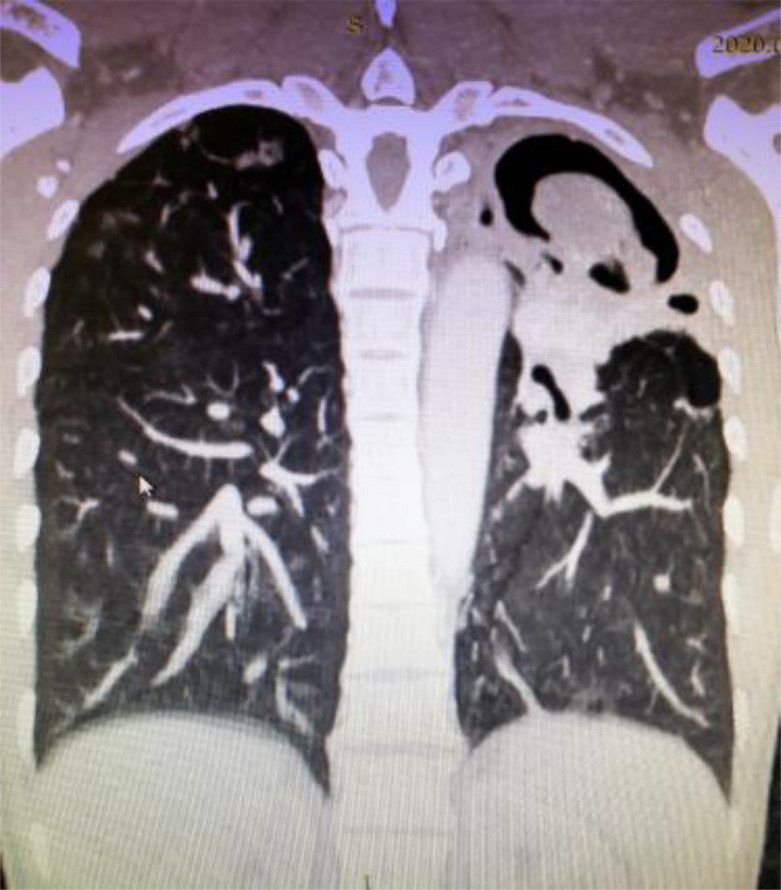
Figure 2: Left upper lobe intra-cavitary mass, with an air crescent sign

An initial plain chest x-ray was done for 70(97.2%) patients and demonstrated a cavitary lesion with a hyperdensity within it. Subsequently, CT scan was done for all patients, and the diagnosis of PA was made by identifying air crescent sign (Figure 1,2). CT scan was also used to further characterize the lesion, to assess the involved site and lobe of the lung and to assess the concomitant disease of the lung. Almost all patients had upper lobe aspergilloma, 71(98.6%), with only one (1.4%) exception who had isolated lower lobe lesion.

When the performance status of the patient was doubtful, the pulmonary function test was used in 17(23.6%) patients that help a better understanding of the general condition. For suspected malignant cavitary lesion, bronchoscopy and BAL cytology were done on 12(16.7%) patients which demonstrated fungal hyphae. Due to the lack of test reagent, the serologic examination to identify possible fungal infection was not done. During surgery, a ball of fungus was demonstrated in all patients, and histologic examination was made for only 11(15.3%) malignant suspected specimens and it demonstrated only A. fumigatus.

### Operative procedures and follow-up

Double lumen Endo-tracheal intubation was used in 46(63.9%) patients. The rest, 26(36.1%) patients, were operated with single-lumen endotracheal tubes. The left lung and right lung were affected in 45(62.5%) and 27(37.5%) patients respectively. In all cases, the pleural surface was found extensively adherent to the chest wall and was difficult to dissect. The type of procedures done was Lobectomy, 32(44.4%), pneumonectomy, 12(16.7%), Bi-lobectomies, 7(9.7%), and cavernostomy, 21(29.2%). The estimated average intra-operative blood loss was 745 +/−307ml (range 300–1600ml) (Table 2).

**Table 2:** Analysis of the location of aspergilloma, affected lobe, type of endotracheal tube used, type of surgery, the estimated blood loss and its relation to Postoperative complications done at Tikur Anbessa Specialized Hospital and Menillik the 2nd Hospital from Nov 30,2014–Nov 30, 2019

Postoperative complications						
	No (%)	Yes (%)	Total (%)	P-Value^1^	AOR	95%CI
Location						
- Right	15(30)	13(59)	28(38.8)	0.022	0.297	0.105–0.842
- Left	35(70)	9(40.9)	44(61.1)			
Affected lobe						
- Upper	50(100)	21(95.5)	71(98.6)	-	-	-
- Middle	0(0)	1(4.5)	1(1.4)			
- Lower	0(0)	0(0)	0(0)			
ETT						
- Single	17(34)	9(40.9)	26(36.1)	0.575	0.744	0.265–2.088
- Double	33(66)	13(59.1)	46(63.9)			
Surgery						
- Pneumonectomy*	4(8)	8(36.4)	12(16.7)	0.011	0.486	0.279–0.847
- Lobectomy	23(46)	9(40)	32(44.4)			
- Bi-lobectomy	5(10)	2(9.1)	7(9.7)			
- Cavernostomy	18(36)	3(13.6)	21(29.2)			
Intraoperative complication
- No	47(94)	16(77.3)	63(88.9)	0.020	5.875	1.31–26.26
- Yes	3(6)	6(22.7)	9(11.1)			
Estimated blood loss	745+/−307ml		0.984	1.000	0.998–1.002

1 Pearson Chi-Square

* reference group used to calculate AOR

During the initial 30 days of postoperative hospital stay, 21(29.1%) patients developed one or more complications. The major postoperative complications seen were prolonged air leak, 7(9.7%), Pneumonia, 3(4.2%), empyema, 7(9.7%), wound infection, 3 (4.2%), and others, 1(1.4). Five (6.9%) patients had significant intraoperative bleeding of which one died due to exsanguinating bleeding from major vessel injury. The other two patients died due to respiratory failure and sepsis which happened after pneumonectomy and lobectomy respectively. The overall mortality within 30 days of surgery was 3(4.2%), and the rest 95.8% of patients were discharged with improvement (Table 3).

**Table 3:** Frequency table of intraoperative and postoperative complications done at Tikur Anbessa Specialized Hospital and Menillik the 2^nd^ Hospital from Nov 30, 2014 – Nov30, 2019

Adverse Outcome	Number	Percent
Intraoperative Complications		
Massive bleeding	5	6.9
Lung lacerations	1	1.4
Death	1	1.4
Others	2	2.8
Total	9	12.5
Postoperative Complications		
Pneumonia	3	4.2
Empyema	7	9.7
Broncho pleural fistula	7	9.7
Wound infection	3	4.2
Death	2	2.8
Others	1	1.4
Total	23	31.9
Grand Total		
No complication	48	66.6
Morbidity	21	29.1
Mortality	3	4.2

Those patients who improved were discharged and followed for a median of 5 months (range 2 weeks to 24 months) at the surgical referral clinic. During the follow-up period, no significant addition of morbidity nor mortality was identified. Few patients were noticed to have received different antifungal treatment.

### Factors predicting adverse outcome

Analysis of the postoperative complications between the type of surgery varied between 9.1% and 40%. It was further evaluated with the chi-square test and a significant difference was detected (p-value=0.011). Patients with pneumonectomy/ lobectomy were most likely to develop complications than those with cavernostomy or Bi-lobectomy. In the bivariate logistic regression models, the affected site of the lung, type of surgery done and the presence of intraoperative complications, were identified to have a significant association with postoperative complications. Postoperative outcome (complication) was significantly affected by the location of the aspergilloma [(P=0.022; AOR=−0.297; 95%CI)], the type of procedure performed [(P=0.011; AOR=−-0.486; 95%CI)] and the presence of intraoperative complications [P=0.020; AOR=5.875; 95% CI).

## Discussion

Pulmonary aspergilloma develops most frequently in residual tuberculosis cavities. The British Thoracic and Tuberculosis Association reported 6% of patients with a healed tuberculosis cavity developing pulmonary aspergilloma within 3 years (17). Many studies also showed pulmonary tuberculosis as the most common pre-existing cavity lesion accounting for 32–45% (28,29). Chen QK from China reported that 71.1% of their patients with PA were treated for tuberculosis (9). In Ethiopia, due to the high incidence of tuberculosis, it is not uncommon to see patients with PA (6). In this study, all patients were previously treated for tuberculosis, and the majority of them placed on a repeated course of anti-tuberculosis treatment. This finding of patients being referred for surgery after treated multiple times for tuberculosis could indicate a wrong effort to resolve PA. This observation in delay referral and possibly wrong treatment of PA requires further study.

Similar to other studies, males are affected more than females (9). Unlike this study, patients with PA in developed countries typically were found in their middle ages. In a series of 18 patients with chronic pulmonary aspergillosis done in the United Kingdom, the median age affected was 59 years (2). According to United Nations data (April 12,2020), the median age in Ethiopian population is 19.5 years. A similar finding of higher proportion of the younger patients with PA was also noticed in this study (17–19).

Clinical presentation of PA ranges from an incidental radiological finding to exsanguinating hemoptysis (20). In our series, cough and hemoptysis were the primary symptoms reported in all patients. Chest pain, SOB, and fever were also reported in half of the cases. A similar study was done in India which reported hemoptysis in 79.16% patients (21). Bleeding during pulmonary aspergilloma usually arises from the bronchial arteries, and it stops spontaneously (22,23). However, when the cavity erodes into the intercostal vessel, the hemoptysis is severe and is unlikely to stop.

All fungal balls in this series were found in the upper lobe. This is because the healed tuberculosis cavities in the upper lobe serve as a good nidus for colonization with A. fumigatus. Many studies reported a similar association (range 13%–89%) (21–23).

Even though HIV infection is a common problem in our society, 73.6% of the patients in this study were not tested for HIV infection. Among those tested, only 2/19 were tested positive. Similar studies showed that since the introduction of potent ART, the incidence of aspergilloma in HIV-infected patients is low. In a review of 342 cases of pulmonary aspergillosis and invasive disease with AIDS, only 14 patients were diagnosed with aspergillomas (12). In this study, a low association of PA with another disease like diabetes mellitus was also identified.

The surgical management of pulmonary aspergilloma is challenging and controversial. No double-blind, placebo-control or randomized trials have been undertaken. According to Shakil et al (24), among the 30 patients operated for PA, 23 were symptomatic and were treated with preoperative antifungal treatment. In this study, although all patients were symptomatic, they were operated without receiving preoperative antifungal treatment.

In 2008, a guideline for the treatment of aspergillosis was developed by the Infectious Diseases Society of America. The guideline recommends an approach to therapy by distinguishing ‘simple aspergilloma’ from the more complex forms of ‘chronic cavitary pulmonary aspergillosis’ and ‘chronic fibrosing pulmonary aspergillosis’ (13). Nevertheless, such kind of diagnostic and therapeutic approach was not followed in the management of our patients.

Surgical treatment for simple aspergilloma is done to prevent or treat potentially life-threatening complications, which usually is curative (14,15). The role of antifungal treatment for simple aspergilloma is controversial. Many patients, such as those who are asymptomatic and have stable radiographic findings over many months, may not require therapy (14). However, if surgery is indicated for symptomatic PA, the use of pre-and post-operative antifungal therapy (usually variconazole) is recommended. This is because during operation, most cavities will inadvertently be opened that leads to spillage, which increases the risk of postoperative aspergillosis (14,25,26). The use of postoperative antifungal treatment in this study was found to be inconsistent. Hence, based on the current recommendation, a protocol on the use of antifungal treatment before and after surgery need to be developed.

In contrast to patients with simple aspergilloma, those with chronic cavitary pulmonary aspergillosis and chronic fibrosing pulmonary aspergillosis require life-long antifungal therapy, a practice that was not reported in our cases management (12–14). The evidence regarding the efficacy of long-term antifungal therapy for such diseases was based on small case series and open-label non-comparative studies. According to those studies, Itraconazole and voriconazole are the preferred oral agents. They also reported that discontinuation of therapy could lead to a gradual return of symptoms, manifested with worsening of chest radiography findings, and rising level of Aspergill (14–16). Surgical outcomes for this category of diseases were not as good as those for simple aspergillomas (12,13).

In general, because of the treatment of massive hemoptysis, surgical resection remains the mainstay of treatment for PA. However, no agreed document exists on the extent of lung resection. In this study, patients with pneumonectomy/lobectomy did not have favorable outcomes compared with those cavernostomy/bilobectomy group. This finding is consistent with a previous study (9,19,). Similarly, Shirakusa et al. advocate a limited lung resection (27). However, studies showed that radical resection of the affected areas had effectively improved patient outcomes. Because of this argument, many case series studies advocate a standard thoracotomy and lobectomy to be the preferred surgical procedures (7–11,18–23). In this series, lobectomy was done for 44.4% of patients. Studies recommend pneumonectomy only when the affected lung was destroyed or the remaining lobe was severely fibrotic and small, which cannot expand to fill the chest cavity (7). Cavernostomy was done primarily for either peripheral placed small PA or when the fungal ball lies close to the fissure or hilum and pneumonectomy was not an option because of the patient’s general condition. Similar to other studies, (19–21, 28–32), the postoperative complications related to it were significantly low (P=0.011). Cavernostomy is now considered as a viable treatment alternative even to those that can be submitted to pulmonary resection.

We investigated the risk factors of postoperative complications and short-term observation of patients with PA. Among the study population, 21(29.1%) of the patients developed postoperative complications. At risk-adjusted analysis, socio-demographic characteristics and clinical presentations showed no effect on the incidence of adverse events. However, the affected side of the lung, the type of surgery done, and the presence of intraoperative complications were revealed as the independent predictors of postoperative complications. The reason for the higher postoperative complication in the right lung surgery is unclear.

Previous studies reported operative mortality rates to range from 4 to 22%, (34–37) and postoperative complications of 15–78%, (28) with the current surgical and anesthetic advances; recent reports show an improved outcome (34). Several of such series describe acceptable outcomes with 2 to 5% mortality and 25% overall complication rate (28–35) Overall, the result of this study is equally favorable to other recent results.

Some of the limitations of this study include the following. First the size of the study population is relatively small. Second, because of the study design (retrospective cross-section) selection bias towards selecting more symptomatic and referred patients than asymptomatic and not referred patients is expected. Third, Bronchoscopy and cytology were done for only 16.7% of patients. The diagnosis was primarily based on the radiologic and intraoperative findings. Hence, some diseases like actinomycosis, nocardiosis, intracavitary hematoma, and adenocarcinoma, could mimic PA. (32). Fourth, because of the extended time period between first incidence of tuberculosis and subsequent development of aspergilloma, it is difficult to ascertain the exact time which may result in a recall bias. And fifth, the duration of follow-up was relatively short, and the long-term outcome of surgery is not known. Therefore, a well-designed prospective cohort study is recommended to confirm the association between different variables and the immediate and long-term adverse outcome.

Finally, the study represents one of the largest surgical experience in Sub-Saharan countries with a resource-limited setup. An outcome, which was comparable with many international studies done in developed countries was achieved. It is, therefore, logical to recommend operative resection of the involved area of the lung with a thorough preoperative assessment and a careful evaluation of the risk/benefit ratio is made.

In conclusion, since pulmonary aspergilloma represents a spectrum of different disease stages, and the risks and benefits of medical and surgical therapy vary with the manifestations of the disease and the patient's pulmonary status, the approach to therapy should be individualized. To improve our patients’ outcome, treating aspergilloma patients in line with those of the 2008 Infectious Diseases Society of America guidelines is recommendable (13).

## References

[1] Judson MA, Stevens DA (2001). The treatment of pulmonary aspergilloma. Curr Opin Investig Drugs.

[2] Denning DW, Rinoiotis K, Dobrashian R, Sambatakou H (2003). Chronic cavitary and fibrosing pulmonary and pleural aspergillosis: case series, proposed nomenclature change, and review. Clin Infect Dis.

[3] Smith NL, Denning DW (2011). Underlying conditions in chronic pulmonary aspergillosis including simple aspergilloma. Eur Respir J.

[4] Sonnenberg P, Murray J, Glynn JR (2000). Risk factors for pulmonary disease due to culture positive M. tuberculosis or non-tuberculosis mycobacteria in South African gold miners. Euro Respir J.

[5] Denning DW, Pleuvry A, Cole DC Global burden of chronic pulmonary aspergillosis as a sequel to pulmonary tuberculosis.

[6] Tafese B. Tufa, Denning DW (2009). The Burden of fungal infection in Ethiopia, Review. J. Fungi.

[7] Brik A, Salem AM, Kamal AR (2008). Surgical outcome of pulmonary aspergilloma. Eur J Cardiothorac Surg.

[8] Chen JC, Chang YL, Luh SP (1997). Surgical treatment for pulmonary aspergilloma: a 28year experience. Thorax.

[9] Chen QK, Jiang GN, Ding JA (2012). Surgical treatment for pulmonary aspergilloma: a 35-year experience in the Chinese population. Interact Cardiovasc Thorac Surg.

[10] Jewkes J, Kay PH, Paneth M (1983). Pulmonary aspergilloma: analysis of prognosis in relation to hemoptysis and survey of treatment. Thorax.

[11] Arvind Kumar, Belal Bin Asaf, Harsh Vardhan Puri et al (2017). Video-assisted thoracoscopic surgery for pulmonary aspergilloma. Lung India.

[12] Mylonakis E, Barlam TF, Flanigan T, Rich JD (1998). Pulmonary aspergillosis and invasive disease in AIDS: review of 342 cases. Chest.

[13] Walsh TJ, Anaissie EJ, Denning DW (2008). Treatment of aspergillosis: clinical practice guidelines of the Infectious Diseases Society of America. Clin Infect Dis.

[14] Jain LR, Denning DW (2006). The efficacy and tolerability of voriconazole in the treatment of chronic cavitary pulmonary aspergillosis. J Infect.

[15] Sambatakou H, Dupont B, Lode H, Denning DW (2006). Voriconazole treatment for subacute invasive and chronic pulmonary aspergillosis. Am J Med.

[16] Felton TW, Baxter C, Moore CB (2010). Efficacy and safety of posaconazole for chronic pulmonary aspergillosis. Clin Infect Dis.

[17] British Thoracic and Tuberculosis Association (1970). Aspergilloma in residual tubercular cavities – The results of a survey. Tubercle.

[18] Nonga   (2018). Complex pulmonary aspergilloma: Surgical challenges in a third world setting, Surgery Research and Practice.

[19] Igai H.; Nagashima T (2012). Pulmonary aspergilloma treated by limited thoracoplasty with simultaneous cavernostomy and muscle transposition flap. Ann Thorac Cardiovasc Surg.

[20] Rergkliang C, Chetpaophan A, Chittithavorn V, Vasinanukorn P (2004). Surgical management of pulmonary cavity associated with fungus ball. Asian Cardiovasc Thorac Ann..

[21] Mohapatra Biswajeet, Sivakumar Poornima, Bhattacharya Subhankar, Dutta Santanu (2016). Surgical treatment of pulmonary aspergillosis: a single center experience. Lung India.

[22] Babatasi G, Massetti M, Chapelier A, Fadel E, Macchiarini P, Khayat A (2000). Surgical treatment of pulmonary aspergilloma: Current outcome. J Thorac Cardiovasc Surg..

[23] Park CK, Jheon S (2002). Results of surgical treatment for pulmonary aspergilloma. Eur J Cardiothorac Surg..

[24] Farid (2013). Results of chronic pulmonary aspergillosis, optimal antifungal therapy and proposed high risk factors for recurrence – a national centers experience. Journal of Cardiothoracic Surgery.

[25] Sapienza Lucas G, Gomes Maria José L, Maliska Carmelindo, Norberg Antonio N (2015). Hemoptysis due to fungus ball after tuberculosis: A series of 21 case treated with hemostatic radiotherapy. BMC Infectious Diseases.

[26] Camuset J, Nunes H, Dombret MC (2007). Treatment of chronic pulmonary aspergillosis by voriconazole in none immune-compromised patients. Chest.

[27] Shirakusa T, Ueda H, Saito T, Matsuba K, Kouno J, Hirota N (1989). Surgical treatment of pulmonary aspergilloma and Aspergillus empyema. Ann Thorac Surg.

[28] Silva   (2014). Complex pulmonary aspergilloma treated by cavernostomy. Rev. Col. Bras. Cir..

[29] Csekeo A, Agócs L, Egerváry M, Heiler Z (1997). Surgery for pulmonary aspergillosis. Eur J Cardiothorac Surg.

[30] Cesar JM, Resende JS, Amaral NF, Alves CM, Vilhena AF, Silva FL (2011). Cavernostomy resection for pulmonary aspergilloma: a 32-year history. J Cardiothorac Surg.

[31] Sagawa M, Sakuma T, Isobe T, Sugita M, Waseda Y, Morinaga H (2004). Cavernoscopic removal of a fungus ball for pulmonary complex aspergilloma. Ann Thorac Surg.

[32] Tseng YL, Wu MH, Lin MY, Lai WW (2000). Intrathoracic muscle flap transposition in the treatment of fibrocavernous tuberculosis. Eur J Cardiothorac Surg.

[33] Guimarães CA, Montessi J, Marsico GA, Clemente AM, Costa AMM, Saito E (2001). Pneumostomia (cavernostomia) no trata-mento da bola fúngica. XII Congresso Brasileiro de Cirurgia torácica, 2001, *Gramado/RS. J Pneumol*.

[34] Lejay A, Falcoz PE, Santelmo N (2011). Surgery for aspergilloma: time trend towards improved results?. Interact Cardiovasc Thorac Surg.

[35] Akbari JG, Kerala P, Neema PK, Menon MU, Neelakhan KS (2005). Clinical profile and surgical outcome for pulmonary aspergilloma: a single center experience. Ann Thorac Surg.

[36] Daly RC, Pairolero PC, Piehler JM, Trastek VF, Payne WS, Bernatz PE (1986). Pulmonary aspergilloma, results of surgical treatment. J Thorac Cardiovasc Surg.

[37] Garvey J, Crastnopol P, Weisz D, Khan F (1977). The surgical treatment of pulmonary aspergillomas. J Thorac Cardiovasc Surg.

